# Effect of biodegradable magnesium foil as an anti-adhesion barrier on the healing of rat Achilles tendon

**DOI:** 10.3389/fbioe.2026.1790167

**Published:** 2026-06-01

**Authors:** Xiaowei Xue, Ting Han, Kai Yan, Jihang Dai, Tianliang Wang, Jingcheng Wang, Jiaxiang Gu

**Affiliations:** 1 The Yangzhou School of Clinical Medicine of Dalian Medical University, Yangzhou, Jiangsu, China; 2 Northern Jiangsu People’s Hospital Affiliated to Yangzhou University, Yangzhou, China; 3 Department of Orthopedics, Northern Jiangsu People’s Hospital, Yangzhou, China; 4 School of Nursing, Faculty of Medicine, Yangzhou University, Yangzhou, Jiangsu, China; 5 College of Mechanical Engineering, Yangzhou University, Yangzhou, China

**Keywords:** Achilles tendon healing, biodegradable metal, biomaterials, magnesium foil, tendon adhesion

## Abstract

Magnesium is the fourth most abundant inorganic element in the human body, critical for skeletal development. Biodegradable magnesium materials have excellent biocompatibility and unique bioactivity, widely studied in orthopedics. Thus, we used a rat Achilles tendon injury model to evaluate magnesium foil s effects on adhesion, inflammation, and healing. Results showed magnesium foil effectively reduced peritendinous adhesion, alleviated local inflammation, improved collagen organization, and enhanced biomechanical properties, supporting physiological repair. In conclusion, biodegradable magnesium foil is a promising anti-adhesion barrier for tendon injury treatment.

## Introduction

The Achilles tendon, the largest and strongest tendon in the human body, plays a fundamental role in transmitting force from the gastrocnemius and soleus muscles to the calcaneus, enabling essential movements such as walking, running and jumping. Due to its unique anatomical structure and relatively poor vascularity, the Achilles tendon is highly susceptible to injury (Szergyuk et al., 2024; Svedman et al., 2024). Epidemiological studies have reported an incidence of approximately 18–40 cases per 100,000 individuals annually (Svedman et al., 2024). While surgical repair remains the primary treatment for Achilles tendon rupture and significantly reduces the risk of re-rupture, postoperative complications—particularly peritendinous adhesion—can severely impair tendon gliding and functional recovery (Carmont et al., 2022; Pean et al., 2018).

Current strategies for preventing tendon adhesion include pharmacological interventions, biological agents, and biomaterial-based physical barriers (Wang et al., 2023). Agents including dexamethasone, indomethacin, camptothecin, hyaluronic acid, chitosan, vitamin C, and 5-fluorouracil have been investigated for their anti-adhesive properties. Growth factors, including IGF-I, TGF-β, VEGF, PDGF, and bFGF, have also been evaluated. Physical barriers such as nylon, cellophane, silicone membranes, absorbable gelatin sponges, PTFE films, and polyethylene films can mechanically separate tendons from surrounding tissues, but poor permeability and non-degradability limit their clinical application (Chen et al., 2015) and may lead to foreign body reactions, secondary adhesions, ulceration, or even tendon necrosis (Mariani et al., 2019).

Biodegradable metals, particularly magnesium- and zinc-based alloys (Zhang and Ren, 2020), have recently attracted widespread attention in biomedical applications due to their biocompatibility, favorable degradation characteristics, and inherent bioactive properties (Seitz et al., 2015). As highlighted in a recent systematic review, magnesium-based alloys exhibit great potential as reinforcing components in biopolymer composites and surface coatings, with excellent biodegradability and biocompatibility for orthopedic and tissue engineering applications (Verma and Ogata, 2023). In-depth research on the microstructural deformation behavior of magnesium-containing alloys lays a theoretical foundation for the structural design and performance optimization of magnesium-based biomedical implants (Verma et al., 2024). Magnesium, the fourth most abundant cation in the human body, is essential for metabolic processes, membrane stability, and the structural integrity of proteins and nucleic acids (Gröber et al., 2015). Magnesium deficiency is associated with increased osteoclast activity and reduced bone mass (Fatima et al., 2024). While most studies have focused on magnesium’s osteogenic effects, its immunomodulatory and tissue-regenerative activities may also be beneficial for soft tissue repair, including tendon healing (Zhou et al., 2024).

Magnesium-based biomaterials exhibit multiple favorable biological effects in orthopedic scenarios, such as regulating osteogenesis, angiogenesis, and neurogenesis, as well as modulating inflammatory responses (Zhao et al., 2017). Released magnesium ions stimulate osteoblast proliferation and tissue repair (Shan et al., 2022), and their immunomodulatory effects involve regulation of cytokine expression and NF-κB signaling (Sugimoto et al., 2012; Ben Amara et al., 2023). Recent findings suggest that magnesium-based materials may suppress inflammation and fibrosis by downregulating the TLR4/NF-κB pathway and activating VEGF signaling (Ben Amara et al., 2023). Given that excessive inflammation response is central to tendon adhesion formation, magnesium’s immunoregulatory characteristics may offer therapeutic value.

Despite these favorable biological properties, magnesium has not yet been systematically investigated for anti-adhesion use in tendon injury (Zhao et al., 2017; Mir et al., 2018). In this study, magnesium foil was applied as a degradable physical barrier to prevent peritendinous adhesion in a rat partial Achilles tendon injury model. Its effects on adhesion formation, collagen arrangement, inflammatory marker expression, and biomechanical function were assessed (Xu et al., 2025). To our knowledge, this is the first study to evaluate magnesium foil as a tendon anti-adhesion material. The findings provide experimental evidence supporting the potential application of magnesium-based biomaterials in tendon repair.

## Materials and methods

All experimental procedures were conducted in accordance with the NIH Guide for the Care and Use of Laboratory Animals. All *in vitro* and *in vivo* detections, including material-related characterization, histological staining analysis, and tendon biomechanical measurement, were performed following widely accepted laboratory protocols and classic research methods in orthopedic and biomaterial studies. All animal experimental protocols were reviewed and approved by the Animal Ethics Committee of Yangzhou University (Approval No. 202509043).

### Preparation of magnesium foil

High-purity magnesium foil (Suzhou Jingjun New Material Co., Ltd.) was prepared via melt-spinning rapid solidification. To enhance its flexibility and corrosion resistance, the foil was immersed in 40% hydrofluoric acid (Sigma-Aldrich) for 24 h. The surface morphology of the magnesium foil was observed using a scanning electron microscope (SEM, JEOL, Japan), and its elemental composition was analyzed by energy dispersive spectroscopy (EDS, Oxford Instruments, United Kingdom).

### Rat Achilles tendon injury model

Forty-eight Wistar rats (4–6 months old, 350 g) were randomized into three groups. The three groups were designated as follows: Control group: Sham operation (skin incision and suture closure only, without tendon transection or repair). Model group: Partial Achilles tendon transection followed by surgical repair, without magnesium foil implantation. Mg group: Partial Achilles tendon transection and surgical repair, with wrapping of biodegradable magnesium foil around the repair site. Anesthesia was induced with sodium pentobarbital (40 mg/kg) administered via intraperitoneal injection (i.p.), inducing stable and deep anesthesia lasting approximately 1 h, which was sufficient for the completion of all experimental procedures. Postoperative analgesia was provided with buprenorphine (0.05 mg/kg), administered via subcutaneous injection (s.c.). A partial tendon transection was performed 3 mm proximal to the calcaneal insertion and repaired using a modified Kessler technique. The knee was fixed in extension and the ankle in plantarflexion using a Kirschner wire. At the conclusion of the experiment, animals were euthanized with an overdose of sodium pentobarbital (180 mg/kg, i.p.), and death was confirmed by the cessation of respiration and heartbeat, along with the absence of reflexes. All rats underwent bilateral Achilles tendon surgery, and both hindlimbs were used as independent biological specimens.

### *In vitro* cell culture

Cell viability assays were conducted using mouse fibroblast cells (L929 cells). Cells were cultured in Dulbecco’s Modified Eagle Medium (DMEM) supplemented with 10% fetal bovine serum and antibiotics (100 U/mL penicillin and 100 μg/mL streptomycin) at 37 °C in a humidified atmosphere with 5% CO_2_. The culture medium was refreshed three times per week. Upon reaching confluence, cells were harvested using 0.25% trypsin digestion. Finally, samples were transferred to 24-well plates (1 × 10^5^ cells/mL, 100 μL per well) for subsequent experiments.

### Adhesion scoring

Adhesion severity was quantified via gross observation immediately following animal euthanasia to assess the extent and severity of peritendinous scar tissue. Scoring was performed using a modified five-point semi-quantitative grading system, conducted by two (or three) independent, blinded observers, and the average score was recorded. The criteria are defined as follows: 0, no adhesion (no adhesions observed around the tendon); 1, mild adhesion (easily separable); 2, moderate adhesion (requiring blunt dissection); 3, severe adhesion (dense and firm, requiring sharp dissection). This scoring method provides an objective and reproducible quantitative basis for evaluating the anti-adhesion efficacy of different treatment groups.

### H&E staining

The tissues were fixed in 4% paraformaldehyde, dehydrated through an ethanol series, and embedded in paraffin wax. Sections of 4–5 μm thickness were cut using a microtome. Prior to staining, the sections were deparaffinized and rehydrated. Hematoxylin was then used to stain the cell nuclei blue/purple. After a brief wash, Eosin stained the cell cytoplasm and extracellular matrix pink. The slides were subsequently dehydrated again and mounted for microscopic observation and pathological analysis.

### Masson’s trichrome staining

After standard deparaffinization and rehydration, tissue sections were fixed in Bouin’s solution for 1 h. Sections were then treated with Weigert’s iron hematoxylin to stain the nuclei black. This was followed by Biebrich Scarlet-Acid Fuchsin to stain the cytoplasm red. To differentiate, sections were treated with Phosphotungstic/Phosphomolybdic acid. Finally, Aniline Blue or Light Green was applied, resulting in collagen fibers staining blue or green. This method effectively highlights the degree of fibrosis and collagen organization in the tendon.

### Immunofluorescence

To evaluate the local inflammatory response and the quality of extracellular matrix remodeling, immunofluorescence staining was performed on the peritendinous tissues. Specifically, the sections were incubated overnight at 4 °C with primary antibodies directed against anti-inflammatory markers (IL-10) and pro-inflammatory cytokines (TNF-α), as well as the principal structural proteins of the tendon matrix, including Type I collagen (COL-1) and Type III collagen (COL-3). Following secondary antibody incubation and nuclear counterstaining, images were captured using a fluorescence microscope. The mean fluorescence intensity (MFI) for each marker was subsequently quantified using ImageJ software (National Institutes of Health, USA). To ensure data accuracy and objectivity, three to five randomly selected high-power fields per section were analyzed.

### Biomechanics

To evaluate the functional recovery and mechanical integrity of the repaired tendons, biomechanical testing was performed using a universal testing machine (Instron, Norwood, MA, USA) equipped with a [e.g., 100 N] load cell. The calcaneus-Achilles tendon-muscle complexes were harvested and securely fixed between two custom-designed clamps to prevent slippage. A preload of [e.g., 0.1N] was applied to stabilize the tissue, followed by tensile testing to failure at a constant loading rate of 10 mm/min. The ultimate load (N), defined as the maximum force sustained before failure, was recorded and analyzed. All tests were conducted under [e.g., saline-moistened] conditions to ensure tissue hydration.

### Statistics

Statistical analysis of the adhesion scores was performed using the Kruskal–Wallis H test followed by Dunn’s multiple comparison test for *post hoc* analysis. All other quantitative data, including biomechanical properties and CCK-8 assay results, were analyzed using one-way analysis of variance (ANOVA). The normality of data distribution and homogeneity of variance were tested prior to statistical analysis. Differences with P < 0.05 were considered statistically significant.

## Results

### Characterization of magnesium foil

**FIGURE 1:**
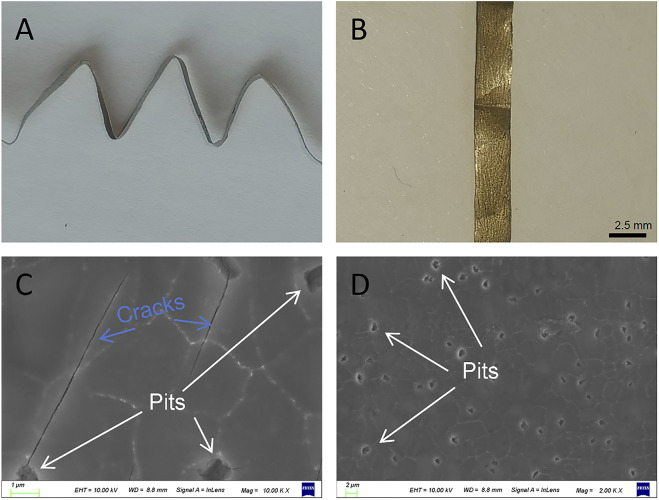
Macroscopic flexibility and surface morphology of the rapidly solidified magnesium foil. **(A)** W-shaped folded foil. **(B)** Flattened foil. **(C)** SEM image of folded foil showing microcracks crossing micro-pits. **(D)** SEM image of as-prepared foil showing uniformly distributed micro-pits.

The magnesium foil is flexible and remains intact after bending, folding and flattening, as shown in Figure 1. SEM images show that the pure magnesium foil prepared by melt-spinning method has a smooth surface. However, many micro pits with a diameter of about 1 μm are uniformly distributed on the surface, which is caused by the surface shrinkage during rapid solidification. Moreover, uniformly distributed microcracks crossing through the micro pits can be observed on the surface of folded magnesium foil. It indicates that the strain due to bending deformation is distributed evenly throughout the strain region and it prevents fracture of sample due to excessive localized strain.

**FIGURE 2:**
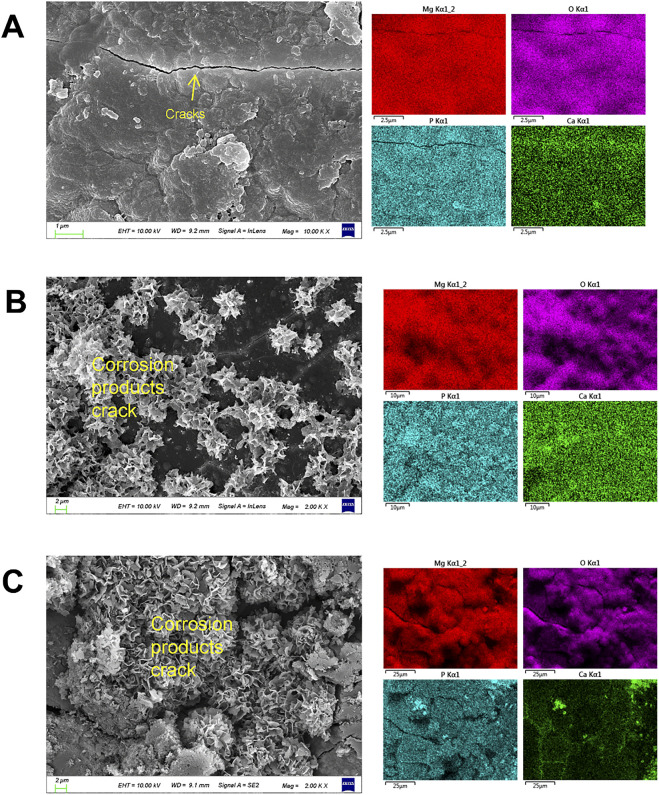
SEM and EDS mapping images of corrosion morphology of rapid solidification magnesium foil. **(A)** after 1 day. **(B)** after 3 days and. **(C)** after 7 days in Hank’s solution at 37 °C.

Figure 2 shows the morphology and elements distribution of corrosion surface after immersion in Hank’s solution. After immersing for 1 day, the corrosion products were deposited and accumulated locally on the surface of the magnesium foil. After 3 days, the corrosion products became thicker, and flower-like corrosion products had formed on the initial corrosion surface. These had a lamellar internal structure typical of magnesium hydroxide lamellar clusters, as verified by EDS mapping. After 7 days, the magnesium foil had corroded severely and broken, with the surface of the residual foil completely covered in corrosion products. The corrosion product layer exhibited obvious cracks, with small calcium-phosphorus compound particles (possibly hydroxyapatite) scattered on the surface.

**FIGURE 3:**
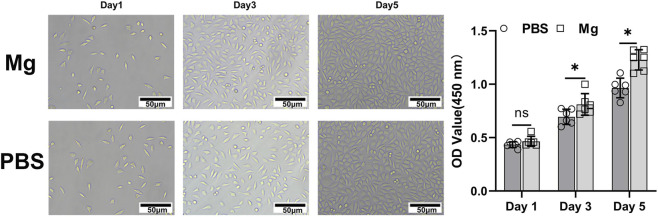
Biocompatibility and proliferation of L-929 mouse fibroblasts cultured with magnesium foil extract. PBS-treated cells served as the control group. The ordinate represents optical density (OD) value measured at 450 nm, and the abscissa represents culture time (days).

For *in vitro* experiments, mouse fibroblast cells (L-929) were co-cultured with magnesium foil extract (prepared in accordance with ISO 10993-12 standard) for 1, 3, and 5 days to observe cellular morphology. As shown in Figure 3, cells cultured in both the magnesium foil extract and the PBS-treated control medium exhibited clear spindle-shaped morphology with actively dividing cells. Notably, the cell density in the magnesium foil group was higher than that in the control group. Cytotoxicity was evaluated using the CCK-8 assay, with the control group consisting of cells cultured in PBS-treated medium. The optical density (OD) values of the magnesium foil group on Day 1 were comparable to those of the control group, indicating no significant difference in cell viability. Over time, cell proliferation increased in both groups, with the magnesium foil group demonstrating a faster proliferation rate compared to the control group. These results suggest that the magnesium foil material is more conducive to cell growth.

### Gross observation of tendon healing

Postoperative observations at 2 and 4 weeks confirmed stable fixation of the Kirschner wires without loosening or dislodgement. During the first postoperative week, one mortality occurred in each of the Control and Mg groups. Additionally, unilateral limb necrosis was noted in one animal from the Mg group, likely due to iatrogenic injury to a major arterial vessel during K-wire fixation. No significant redness, swelling, or exudate was observed around the incision sites in any group. Limb swelling was present across all three groups, with no marked intergroup differences noted.

Rats were sacrificed at 2 and 4 weeks for gross assessment of peritendinous adhesion. Each group originally included 16 rats. At 2 weeks, 14, 16 and 15 valid specimens were obtained in the Control, Model, and Mg groups, respectively. Notably, the Mg group included seven rats with bilateral specimens and one rat with only a single valid limb. At 4 weeks, 16, 16 and 14 specimens were collected in the three groups correspondingly.

All specimens at 2 weeks were used for histological staining and immunohistochemistry, with no biomechanical evaluation. The 4-week specimens were equally divided: half for histological and immunohistochemical analysis, and the other half for biomechanical testing. The biomechanical sample sizes were 8, 8 and 7 in the Control, Model, and Mg groups. All statistical analyses were performed according to the actual available specimen numbers.

**FIGURE 4:**
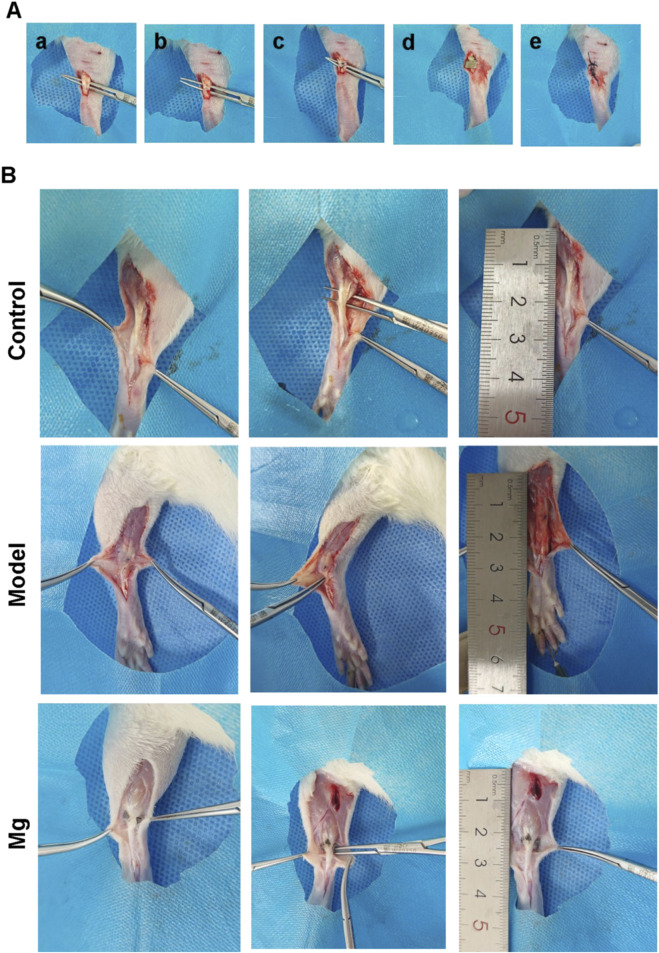
General Procedures and Key Gross Findings in Rat Sample Collection. **(A)** Surgical Procedure for Rat Tendon Healing. **(B)** Gross Morphological Evaluation at 4 weeks Post-Modeling. The experimental cohorts consisted of three distinct groups: the Control group, Model group, and Mg group.

As illustrated in Figure 4A, the surgical procedure was performed as follows: after anesthetizing the rat, a skin incision was made over the Achilles tendon region, followed by blunt dissection to expose the tendon. A precise tenotomy was then performed using a surgical blade. The transected tendon was wrapped with magnesium foil, and the skin was sutured closed. Postoperative immobilization was achieved using Kirschner wires.

Tendon samples were harvested at 2 and 4 weeks post-surgery (Figure 4B). Subsequently, the degree of adhesion was evaluated using the grading system established by Zhang et al. (2011), and the detailed scoring criteria have been fully described in the Methods section. The adhesion scores of each group are summarized in Table 1.

**TABLE 1:** Distribution of adhesion scores at 2 and 4 weeks postoperatively, with intergroup comparisons (P < 0.05).

Adhesion scoring	2 weeks post-op			4 weeks post-op		
	Control	Model	Mg	Control	Model	Mg
0	6	0	0	9	0	0
1	8	0	4	7	0	1
2	0	6	9	0	3	9
3	0	10	2	0	13	4

The tendon adhesion scores at 2 and 4 weeks postoperatively are detailed in Table 1.

At 2 weeks postoperatively, swelling in the bilateral hindlimbs had significantly subsided compared to earlier observations. The tendons and peritendinous tissues were edematous, with evident adhesion to the surrounding tissues. The adhesion scores were 0–1 in the Control group, 2–3 in the Model group, and 1–3 in the Mg group. Among the three groups, the Control group exhibited the least adhesion, followed by the Mg group, while the Model group showed the most severe adhesion.

By 4 weeks postoperatively, all Achilles tendons had healed completely with no cases of re-rupture. Compared with the 2-week time point, adhesion in the Control group showed no tendency to worsen, whereas adhesion in both the Model and Mg groups had increased. The comparative severity among the three groups remained consistent with the observations at 2 weeks.

Comparative analysis revealed that the Model group exhibited severe tendon adhesion and disorganized fiber alignment relative to the Control group. In contrast, the Mg group showed significantly reduced adhesion. Furthermore, a noticeable reduction in the size of the magnesium foil was observed at 4 weeks compared to its initial dimensions, indicating its progressive *in vivo* degradation.

### Biomechanical properties of repaired tendons

To quantitatively evaluate the functional recovery and mechanical strength of the repaired Achilles tendons, a universal tensile testing machine was employed to measure the ultimate tensile force at 4 weeks post-operation.

**FIGURE 5:**
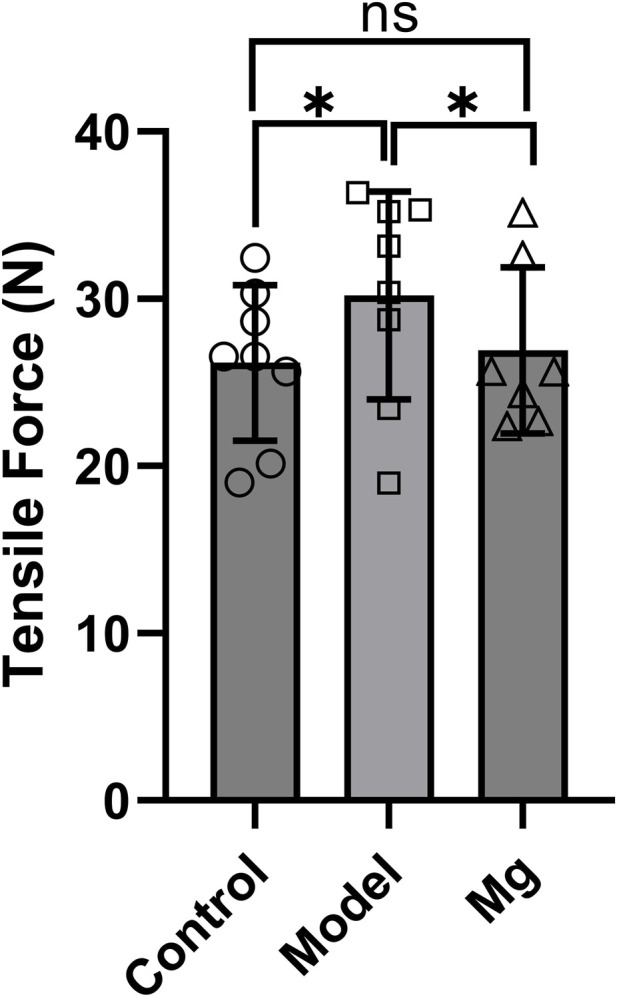
Four-week biomechanical testing showed that the Model group exhibited a pathologically elevated ultimate tensile force due to dense adhesions, while the Mg group successfully restored tensile strength to near-physiological levels, showing no significant difference compared to the Control group.

As illustrated in Figure 5, the Model group exhibited the highest tensile force (approximately 30.1 ± 6.2 N), which was significantly greater than that of both the Control group (26.3 ± 4.8 N) and the Mg group (27.0 ± 5.1 N). This paradoxical increase in the Model group was attributed to the formation of dense, disorganized peritendinous adhesions that physically fused the tendon to surrounding tissues, thereby increasing the total force required for rupture.

Notably, the tensile force in the Mg group was significantly lower than that of the Model group and showed no statistically significant difference compared to the healthy Control group. These findings demonstrate that while the Model group developed a pathological “over-strengthening” due to extensive scar tissue, the application of magnesium foil effectively limited extrinsic tissue ingrowth. Consequently, the Mg group achieved a biomechanical profile that closely approximated the physiological state of healthy tendons, suggesting superior restoration of independent tendon gliding.

### Magnesium foil promotes tendon healing

To investigate the effects of magnesium foil on Achilles tendon healing *in vivo*, we established a rat model of Achilles tendon transection. The animals were randomly divided into three groups: the Control group, the Model group and the Mg group. Tendon samples were collected at 2 and 4 weeks post-surgery, fixed in formalin, embedded in paraffin, and sectioned for hematoxylin and eosin (HE) and Masson’s trichrome staining.

**FIGURE 6:**
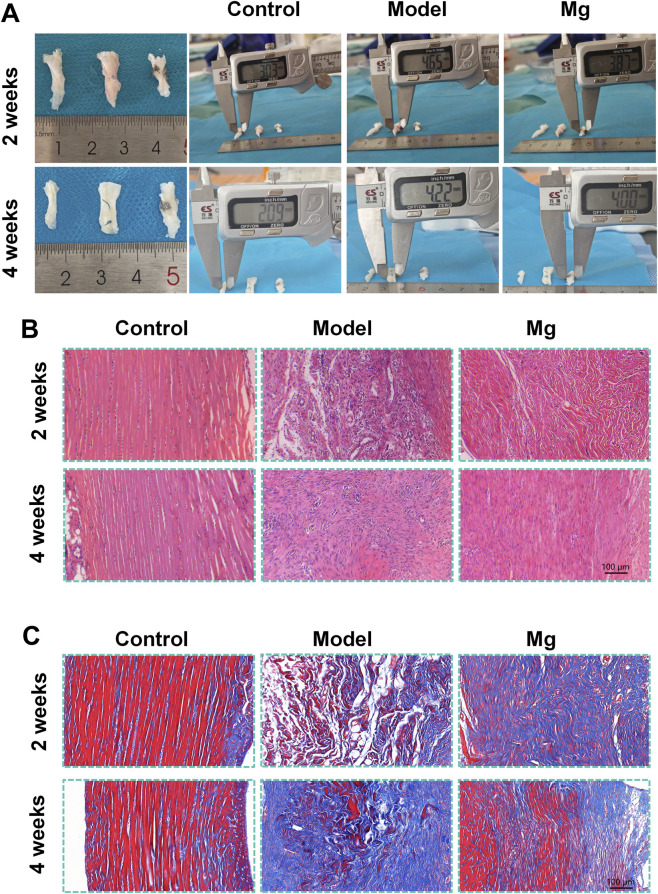
Histological analysis of tendon healing. **(A)** Gross morphology showing reduced thickening and improved tendon appearance in the Mg group compared to the Model group. **(B)** HE staining revealed disorganized collagen fibers, inflammatory infiltration, and fibroblast proliferation in the Model group, while the Mg group exhibited more ordered tissue structure with diminished inflammation and improved collagen alignment. **(C)** Masson’s trichrome staining demonstrated a more organized collagen alignment in the Mg group at the 4-week time point compared to the Model group. Scale bar = 100 μm.

Gross morphological observations (Figure 6A) revealed that compared to the Control group, the Model group exhibited significant tissue thickening and swelling at the healing site, consistent with typical fibrotic scar healing. In contrast, the Mg group displayed a more slender tendon appearance, with morphology and diameter closer to those of the normal Control group, suggesting that magnesium foil intervention effectively improved the quality of tendon healing and suppressed excessive proliferation.

As shown in (Figure 6B), H&E staining of tendon sections from each group was performed to evaluate tendon healing and local inflammatory responses. Our analysis revealed distinct microstructural differences among the three groups. The Control group exhibited tightly arranged, regular and parallel collagen fiber bundles with normal cellular architecture. In contrast, the Model group displayed a characteristic disorganized healing pattern, featuring loose collagen structure, highly irregular fiber alignment, extensive fibroblast proliferation, inflammatory cell infiltration, and increased vascularization—all indicative of scar tissue formation. The Mg group demonstrated significant histological improvement, with more organized collagen fiber arrangement, reduced inter-fiber spacing, markedly diminished inflammatory cell infiltration, and fibroblast density and morphology approaching normal tissue levels. Collectively, these histological findings indicate that magnesium foil promotes functional tendon healing through structurally ordered regeneration rather than scar-mediated repair.

This observation was further supported by Masson’s trichrome staining results (Figure 6C). While the Mg group showed collagen organization similar to the Model group at the 2-week time point, by 4 weeks, the Mg group exhibited substantially improved collagen alignment and maturity compared to the Model group, demonstrating the progressive beneficial effect of magnesium foil on tendon remodeling.

### Magnesium foil primarily facilitates Achilles tendon healing by modulating the local inflammatory microenvironment

**FIGURE 7:**
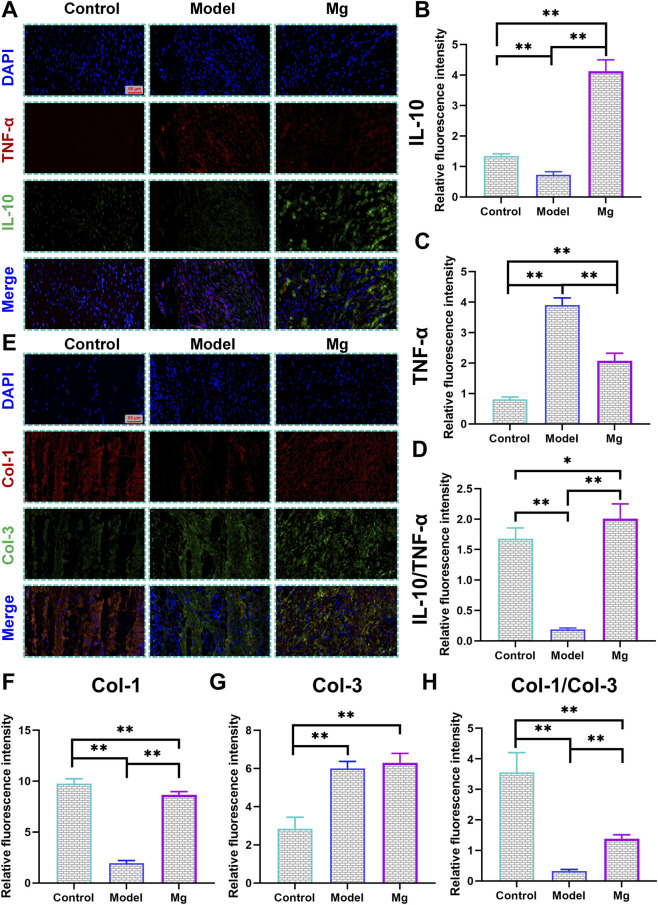
Expression of Collagen and Inflammatory Factors in Tendon Sections. **(A)** Immunofluorescence staining of IL-10 and TNF-α in the Control, Model, and Mg groups. **(B–D)** Quantitative analysis of the fluorescence intensity for these inflammatory factors. **(E)** Representative immunofluorescence images showing Collagen Type I (Col-1) and Collagen Type III (Col-3) expression in the Control, Model, and Mg groups. **(F–H)** Quantitative analysis of the Immunofluorescence staining intensity for Col-1 and Col-3. Scale bar = 50 μm. P < 0.05 was considered statistically significant.

During the early phase of tendon healing, the inflammatory response plays a critical role in the repair process. A moderate inflammatory reaction facilitates tendon regeneration, whereas excessive inflammation adversely affects healing outcomes. As a key anti-inflammatory cytokine, elevated IL-10 expression can suppress excessive inflammation, thereby maintaining a conducive microenvironment for tendon repair. Conversely, TNF-α, a primary pro-inflammatory cytokine, exerts opposing effects by promoting inflammatory cascades. Thus, modulating their expression—by inhibiting TNF-α and enhancing IL-10—is of considerable therapeutic importance. Immunofluorescence staining at 2 weeks post-modeling revealed that the Mg group exhibited a lower inflammatory response compared with the Model group, reaching a level comparable to that of the Control group (Figures 7A–D). This suggests that magnesium foil can mitigate excessive inflammation and thereby support tendon healing.

Collagen Type I (Col-1), the predominant collagen in healthy and mature tendons, constitutes approximately 95%–98% of the total collagen content in normal tendon tissue. In contrast, Collagen Type III (Col-3), though essential in the later stages of healing, may lead to fibrotic scar formation and impaired tendon function when overexpressed. Immunofluorescence analysis of tendon sections at 4 weeks demonstrated that compared with the Control group, Col-1 expression was significantly reduced in the Model group but restored to an appropriate level in the Mg group (Figures 7E–H). In contrast, Col-3 expression exhibited an inverse trend across the groups. These findings indicate that magnesium contributes to improved tendon healing by promoting Col-1 deposition and suppressing excessive Col-3 expression, thereby reducing scar formation and supporting functional recovery.

## Discussion

The present study confirmed that magnesium foil application markedly alleviated peritendinous adhesion and improved tendon healing quality. Apart from its potential role as a physical barrier to block the ingrowth of surrounding granulation tissue and fibroblasts, magnesium foil may also facilitate tendon repair by modulating local inflammatory microenvironment and regulating collagen remodeling.

It is important to emphasize that the advantages of magnesium (Mg) foil are not limited to its physical barrier function. Compared with other common anti-adhesion materials, such as polytetrafluoroethylene (PTFE), silicone sheets, and hyaluronic acid (HA) gels, Mg foil also provides unique immunomodulatory benefits (Xu et al., 2025; Ben Amara et al., 2023). The local release of magnesium and its degradation products has been shown to modulate inflammatory responses and cytokine expression, which may further optimize the healing process and suppress the excessive formation of scar tissue (Chen et al., 2021). Therefore, Mg foil serves as a “dual-action” anti-adhesion material, possessing both physical isolation and bioactive regulatory capabilities, giving it significant application potential in the field of tendon repair.

Tendon healing progresses through three overlapping phases: inflammation, proliferation (matrix deposition), and remodeling (collagen maturation). Modulating the initial inflammatory milieu is critical for mitigating early-stage adhesions. At 2 weeks post-operation, our data indicate that magnesium (Mg) foil effectively modulates the early inflammatory response, which is consistent with the findings of Xia et al. regarding the inhibition of the TLR4/NF-κB pathway and activation of VEGF signaling (Xia et al., 2017). Thus, Mg foil fosters a favorable immune microenvironment that supports subsequent repair.

Importantly, Mg-mediated modulation achieves a “delicate balance” unlike traditional NSAIDs. While early NSAID administration can compromise mechanical properties by over-suppressing essential healing triggers (Gunaydin and Bilge, 2018), Mg foil appears to attenuate excessive fibrosis and adhesion without impairing the fundamental regenerative process.

This study demonstrates that the application of magnesium (Mg) foil significantly upregulates the expression of COL-1 while concurrently downregulating COL-3 levels in repaired tissues (Lopes Silva et al., 2020). The quality of tendon healing is highly dependent on the restoration of the COL-1/COL-3 ratio. During the early stages of healing, excessive deposition of COL-3 leads to increased granulation tissue and scar formation, which serves as the structural basis for tendon adhesion. The elevation of COL-1 concentration marks a transition from disorganized fibrous tissue to a functional, mature tendon structure. Mg ions likely promote this maturation by optimizing local fibroblast activity, thereby enhancing structural integrity while preventing adhesions.

The recovery of biomechanical properties is considered the gold standard for evaluating the success of anti-adhesion materials (Wang et al., 2025). Our results show that the Mg group exhibited a failure load comparable to the Control group. The failure load directly reflects the resistance to rupture at the repair site, and this functional improvement is attributed not only to the optimized collagen arrangement but also to the ability of the Mg foil to isolate the tendon from extrinsic healing processes and maintain an ideal environment for intrinsic repair.

It is noteworthy that the immunomodulatory effects of magnesium differ significantly between bone and soft tissues (Li et al., 2024). The homeostasis of bone environments is maintained by the balance between osteogenesis and osteoclastogenesis (Zhang et al., 2025; Tao et al., 2022). Mg primarily functions by activating osteogenic signals (such as Wnt/β-catenin) and inhibiting osteoclast activity (Kang et al., 2022; Shen et al., 2021). In peritendinous soft tissues, however, the core mechanism lies in the suppression of excessive inflammatory responses. This disparity stems from differences in cellular composition within the tissue microenvironments. Soft tissues are highly sensitive to Mg ion release, which regulates the polarization of macrophages from an M1 to an M2 phenotype (Sheng et al., 2025; Cheng et al., 2022), the latter of which is the key to mediating inflammation and anti-inflammatory processes (Shi et al., 2022; Qian et al., 2023; Shang et al., 2026; Wang et al., 2026; Cai et al., 2025; Qi et al., 2024). This tissue-specific immunoregulation is key to preventing adhesions without inducing excessive hyperplasia.

Despite these encouraging results, several limitations remain. First, this study lacked a quantitative histological scoring system, which would provide a more objective assessment of adhesion severity. Second, the observation period was relatively short, leaving the long-term biological effects of the Mg foil after complete degradation unexplored. Finally, the study focused on phenotypic observations and lacked deep mechanistic assays, such as gene knockout or pathway blockade experiments, to verify specific molecular signaling.

In summary, this study provides a proof-of-concept for using Mg foil as a functional anti-adhesion barrier. Beyond the scope of this work, the findings open new avenues for intelligent biodegradable implants in tendon repair. Future research perspectives are outlined as follows. First, it is essential to systematically elucidate Mg^2+^-mediated biological effects, with an emphasis on the regulatory mechanisms of fibrosis-associated signaling cascades, including the TGF-β/Smad and TLR4/NF-κB pathways. Second, refined degradation kinetic modeling and surface modification of Mg-based materials should be combined to achieve tailored degradation behavior that precisely matches the critical time window of tendon remodeling and healing. Third, systematic parameter optimization, such as screening appropriate Mg foil thickness, will help balance mechanical stability and biocompatibility. Additionally, further efforts are required to promote the clinical translatability of this strategy in complex multi-tissue injury repair.

## Conclusion

Magnesium foil reduced tendon adhesion, improved collagen fiber alignment, and enhanced biomechanical strength in a rat Achilles tendon injury model. These benefits may arise from its physical barrier function, controlled degradation behavior, and modulation of the inflammatory microenvironment. Optimization of material properties and mechanistic studies are warranted. Magnesium foil shows strong potential as a biodegradable anti-adhesion material for tendon repair and provides a foundation for future orthopedic applications.

## Data Availability

The original contributions presented in the study are included in the article/supplementary material, further inquiries can be directed to the corresponding authors.
